# Suppression of the invasive plant mile-a-minute (*Mikania micrantha*) by local crop sweet potato (*Ipomoea batatas*) by means of higher growth rate and competition for soil nutrients

**DOI:** 10.1186/s12898-014-0033-5

**Published:** 2015-01-28

**Authors:** Shicai Shen, Gaofeng Xu, David Roy Clements, Guimei Jin, Aidong Chen, Fudou Zhang, Hisashi Kato-Noguchi

**Affiliations:** Agricultural Environment and Resource Research Institute, Yunnan Academy of Agricultural Sciences, Kunming, 650205 Yunnan China; Biology Department, Trinity Western University, 7600 Glover Road, Langley, V2Y 1Y1 British Columbia Canada; Department of Applied Biological Science, Faculty of Agriculture, Kagawa University, Miki, 761-0795 Kagawa Japan

**Keywords:** Sweet potato, Mile-a-minute, Competition interactions, Soil nutrients, Biological control

## Abstract

**Background:**

There are a variety of ways of increasing crop diversity to increase agricultural sustainability and in turn having a positive influence on nearby natural ecosystems. Competitive crops may provide potent management tools against invasive plants. To elucidate the competitive mechanisms between a sweet potato crop (*Ipomoea batatas*) and an invasive plant, mile-a-minute (*Mikania micrantha*), field experiments were carried out in Longchuan County of Yunnan Province, Southwest China, utilizing a de Wit replacement series. The trial incorporated seven ratios of sweet potato and mile-a-minute plants in 25 m^2^ plots.

**Results:**

In monoculture, the total biomass, biomass of adventitious root, leafstalk length, and leaf area of sweet potato were all higher than those of mile-a-minute*,* and in mixed culture the plant height, branch, leaf, stem node, adventitious root, flowering and biomass of mile-a-minute were suppressed significantly (P < 0.05)*.* The relative yield (RY) of mile-a-minute and sweet potato was less than 1.0 in mixed culture, indicating that intraspecific competition was less than interspecific competition. The competitive balance index of sweet potato demonstrated a higher competitive ability than mile-a-minute. Except pH, other soil nutrient contents of initial soil (CK) were significantly higher than those of seven treatments. The concentrations of soil organic matter, total N, total K, available N, available P, available K, exchange Ca, exchange Mg, available Mn, and available B were significantly greater (P < 0.05) in mile-a-minute monoculture soil than in sweet potato monoculture soil, and were reduced by the competition of sweet potato in the mixture.

**Conclusions:**

Evidently sweet potato has a competitive advantage in terms of plant growth characteristics and greater absorption of soil nutrients. Thus, planting sweet potato is a promising technique for reducing infestations of mile-a-minute, providing weed management benefits and economic returns from harvest of sweet potatoes. This study also shows the potential value of replacement control methods which may apply to other crop-weed systems or invaded natural ecosystems.

## Background

The conventional agricultural paradigm of reliance on chemical or mechanical control of weeds to maintain yields in monocultures often proves to be unsustainable [1,2]. Thus, in many jurisdictions more holistic agroecosystem management approaches are being implemented that incorporate crop diversity, crop rotation, and concomitant reduced inputs for weed control [3,4]. More holistic management approaches tend to increase crop diversity to improve ecosystem health. For example, involving alternative crops in a rotation introduces greater crop diversity. Rotating crops has been found to reduce both the economic impact and diversity of weeds [5,6]. These crop rotation benefits are frequently accounted for by lack of a buildup of problem weeds associated with particular crop as well as rotation of control methods associated with particular crops. Furthermore, an increase in crop diversity has numerous other benefits including improved soil properties, microbial diversity and advantages in terms of insect or fungal pest management as the more diverse agroecosystem takes on more characteristics in common with natural ecosystems [2,6]. There are several other established ways of increasing agroecosystem diversity such as cover cropping, intercropping or diversifying the soil environment through composts or manures [3].

One relatively novel means of increasing crop diversity while improving weed management is by deliberately introducing an alternative crop that is known to be highly competitive with a weed that is difficult to control. Replacement control with high value species (e.g., local food, native species and/or cash crops) recently has emerged as a viable option for management of invasive alien plant species [7-10]. Although essentially a type of crop rotation, the difference is that the competitive crop itself is the means of weed control in the case of replacement control. As a potential alternative to traditional bio-control which generally employs insects or pathogens, replacement control relies on growth advantage of one or more plants to suppress exotic plants, simultaneously reducing damage caused by the invasive species and improving local natural ecosystem health by reducing the potential for invasive plants to spread beyond agricultural fields [10,11]. Compared with mechanical or chemical control methods, replacement control is generally considered more secure, economical, ecological, and sustainable [10]. Replacement control research has recently focused on screening native species for competitiveness, mechanisms of competition, and natural ecosystem restoration effects [9,12,13].

Mile-a-minute (*Mikania micrantha* H.B.K.: Asteraceae), a perennial herb or semi-woody vine, is native to Central and South America [14]. Considered one of the ten worst weeds and the 100 worst invasive alien species in the world [15], the range of mile-a-minute now includes tropical Asia, parts of Papua New Guinea, Indian Ocean islands, Pacific Ocean islands, and Florida in the U.S. [14,16]. In China, the earliest record of mile-a-minute was from 1884 when it was cultivated at Hong Kong Zoological and Botanical Gardens [14]. Its naturalization in Hong Kong dates to 1919 or earlier, and it is believed to have begun expanding into other areas during the 1950s [14], and now it widely distributed in Guangdong, Yunnan, Hainan, Guangxi and Hong Kong [14,17]. The vine has invaded a broad range of farming systems and forest lands, causing serious economic and environmental impacts [14,18,19]. According to Zhong *et al*. [20], the economic impact of mile-a-minute on natural areas alone amounted to more than several hundreds of millions of dollars in China per year.

In order to control mile-a-minute, extensive research has been conducted on mechanical removal, chemical control, biological control, and ecological control over the past two decades [14,21-25]. Nevertheless, due to high capacity for both asexual and sexual reproduction and morphological plasticity [26], high compensation capacity [27], and rapid adaptive evolution [28], no single control method can effectively alleviate the damage caused by the mile-a-minute, and more comprehensive prevention and control measures must be adopted. Replacement control technology, utilizing plant competition, represents a promising component of a more holistic, integrated management strategy. Competition between locally available plants and mile-a-minute has recently been investigated in China [9,29-31]. In 2006 and 2007, sweet potato [*Ipomoea batatas* (L.) Lam.: Convolvulaceae], an important locally grown cash crop native to the American tropics, was observed to inhibit mile-a-minute growth in invaded farming communities in Longchuan County [30], but the competitive mechanisms involved have not been elucidated.

To gain a better understanding of the competitive effects of sweet potato on control of mile-a-minute and associated soil nutrient dynamics of invaded communities in Yunnan Province, Southwest China, we conducted a set of field experiments in Longchuan County where mile-a-minute causes serious economic damage and sweet potato is commonly cultivated. The main objective of this study was to examine competitive mechanisms and soil interaction between sweet potato and mile-a-minute, in order to provide a scientific basis for setting up an effective management method utilizing ecological control techniques for mile-a-minute in the field. Lessons learned from this potential replacement crop system may be applied to other crop-weed combinations in pursuit of more holistic management approaches utilizing plant competition and crop diversification.

## Results

### Plant growth

Comparing growth in monoculture, sweet potato attained twice as much biomass as mile-a-minute; 53.27 ± 0.73 g for sweet potato vs. 25.18 ± 1.35 g for mile-a-minute (Table 1). In mixed culture, the total shoot length (main stem + branch length) and branch length of mile-a-minute were significantly suppressed, and the inhibition rates (except branch length for a ratio of sweet potato to mile-a-minute of 1:3) were significantly higher (P < 0.05) than those of sweet potato with decreasing proportions of mile-a-minute (Table 1). With proportional increases in sweet potato, the main stem length of mile-a-minute was highly suppressed; for ratios of sweet potato to mile-a-minute of 3:1 and 2:1 total shoot length was much greater for sweet potato*;* even at a 1:3 ratio, mile-a-minute shoots were significantly shorter than in monoculture (P < 0.05).

**Table 1:** **Plant growth comparison of sweet potato (** ***Ipomoea batatas*** **) and mile-a-minute (** ***Mikania micrantha*** **) under mono and mixed culture conditions**

**Variables**		**Ratios (sweet potato: mile-a-minute)**						
		**4:0**	**3:1**	**2:1**	**1:1**	**1:2**	**1:3**	**0:4**
Total shoot Length (cm)	sweet potato	802.55 ± 21.12a	729.57 ± 19.41b	713.92 ± 17.43b	668.04 ± 15.74c	588.70 ± 14.55d	519.64 ± 10.51e	-
	mile-a-minute	-	460.61 ± 15.15e	466.99 ± 14.42e	680.32 ± 18.68d	845.31 ± 22.84c	1282.43 ± 26.63b	1426.84 ± 34.99a
Main stem length (cm)	sweet potato	335.50 ± 12.46a	323.06 ± 9.35ab	318.17 ± 10.45b	255.68 ± 8.59c	241.54 ± 8.02d	214.66 ± 7.24e	-
	mile-a-minute	-	209.57 ± 10.13d	206.94 ± 9.46d	255.15 ± 9.36c	329.26 ± 8.45b	435.40 ± 9.84a	431.33 ± 13.38a
Total branch length (cm)	sweet potato	467.05 ± 15.25a	406.51 ± 11.54b	395.75 ± 10.36b	412.36 ± 12.31b	347.16 ± 9.88c	304.98 ± 5.69d	-
	mile-a-minute	-	251.04 ± 9.26e	260.05 ± 10.95e	425.17 ± 15.23d	516.05 ± 17.58c	847.03 ± 20.14b	995.51 ± 28.36a
Branch number	sweet potato	10.5 ± 0.4c	13.4 ± 0.6a	13.3 ± 0.5a	11.4 ± 0.5b	11.9 ± 0.5b	10.4 ± 0.5c	-
	mile-a-minute	-	12.6 ± 0.5d	12.5 ± 0.6d	15.4 ± 0.8c	16.2 ± 0.7c	23.8 ± 0.9b	25.2 ± 1.2a
Internode length (cm)	sweet potato	6.75 ± 0.04a	6.78 ± 0.03a	6.53 ± 0.04b	5.78 ± 0.07d	5.89 ± 0.04c	5.24 ± 0.05e	-
	mile-a-minute	-	7.74 ± 0.15c	10.78 ± 0.12a	7.65 ± 0.36c	8.81 ± 0.13b	10.75 ± 0.19a	10.54 ± 0.14a
Leafstalk length (cm)	sweet potato	17.18 ± 0.51a	16.82 ± 0.29a	14.70 ± 0.22b	14.01 ± 0.31c	13.98 ± 0.25c	13.71 ± 0.26c	-
	mile-a-minute	-	5.68 ± 0.16e	6.13 ± 0.11d	6.57 ± 0.08c	6.76 ± 0.12b	7.08 ± 0.11a	7.06 ± 0.12a
Leaf area (cm^2^)	sweet potato	101.25 ± 1.78a	96.84 ± 1.68b	90.08 ± 1.05c	88.78 ± 0.55c	85.82 ± 0.48d	83.68 ± 0.46e	-
	mile-a-minute	-	12.45 ± 0.12f	13.23 ± 0.09e	14.36 ± 0.35d	16.41 ± 0.36c	17.05 ± 0.45b	21.21 ± 0.25a
Flower number per shoot	sweet potato	24.1 ± 0.7c	27.3 ± 0.9a	26.8 ± 0.7ab	26.3 ± 0.5ab	25.7 ± 0.9b	23.4 ± 0.4c	-
	mile-a-minute	-	420.5 ± 25.4e	515.9 ± 30.1d	755.2 ± 33.7c	731.5 ± 32.1c	1150.2 ± 38.5b	1506.4 ± 65.7a
Adventitious root weight (g)	sweet potato	1.25 ± 0.06b	1.36 ± 0.04a	1.39 ± 0.04a	1.05 ± 0.02c	1.04 ± 0.02c	0.99 ± 0.02c	-
	mile-a-minute	-	0.34 ± 0.04c	0.37 ± 0.03c	0.45 ± 0.03b	0.47 ± 0.02b	0.49 ± 0.03b	0.63 ± 0.04a
Total biomass (g)	sweet potato	53.27 ± 0.73a	46.15 ± 0.23b	42.83 ± 0.43c	34.05 ± 0.36d	31.84 ± 0.22e	23.14 ± 0.19f	-
	mile-a-minute	-	3.35 ± 0.03f	5.26 ± 0.08e	6.35 ± 0.03d	8.29 ± 0.12c	16.15 ± 0.43b	25.18 ± 1.35a

Data are expressed as mean ± standard deviation. The different letters within same row mean significant differences at P<0.05.

The internode length of mile-a-minute was greater in magnitude than that of sweet potato in both mixed and monoculture (Table 1). With decreasing proportions of mile-a-minute, its internode length was reduced to a certain extent by sweet potato but the trend was not too clear. The branch number of mile-a-minute was greater than that of sweet potato in monoculture (Table 1)*.* In mixed culture, the branch number of mile-a-minute was significantly suppressed (P < 0.05), and the inhibition rates were higher than those of sweet potato with decreasing proportions of mile-a-minute.

The leafstalk length and leaf area of mile-a-minute were markedly less than those of sweet potato in all treatments (Table 1). In monoculture, the mean leafstalk length and leaf area of sweet potato were 17.18 cm and 101.25 cm^2^, and the leafstalk length and leaf area of mile-a-minute were only 7.06 cm and 21.21 cm^2^. In mixed culture, mile-a-minute leafstalk length averaged about half that of sweet potato at the highest ratio of mile-a-minute: sweet potato (3:1) and declined to less than one third of sweet potato leafstalk length at the 1:3 ratio. Likewise leaf area of mile-a-minute progressively declined with increasing proportions of sweet potato; even at the 1:3 sweet potato: mile-a-minute ratio, the leaf area of mile-a-minute was just one fifth of the leaf area of sweet potato.

The adventitious root biomass of sweet potato was much greater than that of mile-a-minute in all treatments (Table 1). In mixed culture, the adventitious root biomass of mile-a-minute was significantly suppressed (P < 0.05), and the inhibition rates were higher than those of sweet potato with decreasing proportions of mile-a-minute*.* In monoculture, the total biomass of sweet potato was 2.12 times that of mile-a-minute, and was significantly greater (P < 0.05) than that of mile-a-minute in mixed culture (Table 1). The total biomass of mile-a-minute was significantly suppressed (P < 0.05), and the inhibition rates were higher than those of sweet potato for ratios of sweet potato to mile-a-minute of 3:1, 2:1 1:1, and 1:2. In all treatments, the number of flowers per shoot for mile-a-minute was greater (at least 15 times greater) than for sweet potato (Table 1)*.* Still, in mixed culture, the number of flowers per shoot of mile-a-minute was significantly suppressed (P < 0.05), and the inhibition rates were higher than those of sweet potato with decreasing proportions of mile-a-minute.

### Competitive interactions

The relative yield (RY) of mile-a-minute and sweet potato in different ratios showed that the two plants compete strongly (Table 2). The RY of mile-a-minute and sweet potato was significantly less (P < 0.05) than 1.0 in mixed culture, and only for a ratio of sweet potato to mile-a-minute of 1:3 was the RY of mile-a-minute greater than that of sweet potato*,* showing that the intraspecific competition between two plants was less than their interspecific competition. The relative yield total (RYT) of mile-a-minute and sweet potato was less than 1.0 in mixed culture (ranging from 0.45 to 0.54) indicating that there was competition between the two plants. The competitive balance index (CB) of sweet potato of −0.39 was significantly less than zero (P < 0.05) when grown with mile-a-minute in mixed culture at 1:3 (sweet potato: mile-a-minute), whereas for the other ratios the CB index was greater than zero and the maximum CB index was 1.87. With decreasing proportions of mile-a-minute, the competitiveness of sweet potato increased at a rate exceeding what would be predicted by the increase in relative density.

**Table 2:** **Relative yield, relative yield total and competitive balance index of sweet potato (** ***Ipomoea batatas*** **) and mile-a-minute (** ***Mikania micrantha*** **) in mixed culture**

**Ratios (sweet potato: mile-a-minute)**	**Sweet potato relative yield (RYa)**	**Mile-a-minute relative yield (RYb)**	**Relative yield total (RYT)**	**Competitive balance index (CB) for sweet potato**
3:1	0.87 ± 0.004a**	0.13 ± 0.001d**	0.50 ± 0.001b**	1.87 ± 0.014a**
2:1	0.80 ± 0.008b**	0.21 ± 0.003c**	0.51 ± 0.005b**	1.35 ± 0.008b**
1:1	0.64 ± 0.007c**	0.25 ± 0.001c**	0.45 ± 0.003d**	0.93 ± 0.013c**
1:2	0.60 ± 0.004d**	0.33 ± 0.005b**	0.46 ± 0.002c**	0.60 ± 0.019d**
1:3	0.43 ± 0.004e**	0.64 ± 0.017a**	0.54 ± 0.009a**	−0.39 ± 0.028e**

Data are expressed as mean ± standard deviation. The different letters within same column mean significant differences at P<0.05. The t-test was used to compare each value with 1.0 and 0, ** indicate significant differences at 0.01 level.

### Soil nutrient effects

Soil nutrient characteristics varied significantly (P < 0.05) among the seven different treatments corresponding to the seven ratios of the two species (Table 3). The pH of initial soil (CK) was obviously lower than those of seven treatments, but other soil nutrient contents of initial soil were significantly higher (P < 0.05). In monoculture, the organic matter content, pH, total N content, total K content, available N content, available P content, and available K content of mile-a-minute soil were significantly higher (P < 0.05) than those of sweet potato, and significantly decreased as proportions of sweet potato increased in mixed culture. The total P content of soil from the mile-a-minute monoculture was significantly less than in sweet potato soil (P < 0.05), and increased as the proportion of sweet potato increased in mixed culture.

**Table 3:** **Soil properties (i.e. pH, organic matter, total N, total P, total K, available P, available K, available K, exchangeable Ca, exchangeable Mg, available Cu, available Zn, available Fe, available Mn, and available B) of mile-a-minute (** ***Mikania micrantha*** **) and sweet potato (** ***Ipomoea batata*** **) soils under mono and mixed culture conditions**

**Variables**	**Ratios (sweet potato: mile-a-minute)**							
	**CK**	**4:0**	**3:1**	**2:1**	**1:1**	**1:2**	**1:3**	**0:4**
pH	7.25 ± 0.02e	7.34 ± 0.02d	7.37 ± 0.01c	7.40 ± 0.01b	7.41 ± 0.02b	7.41 ± 0.02b	7.42 ± 0.02b	7.47 ± 0.02a
Organic mater (g/kg)	44.05 ± 0.47a	34.13 ± 0.43d	40.06 ± 0.54c	42.18 ± 0.42b	42.42 ± 0.51b	42.06 ± 0.34b	42.38 ± 0.34b	42.37 ± 0.47b
Total N (g/kg)	1.51 ± 0.03a	1.36 ± 0.02d	1.38 ± 0.02d	1.41 ± 0.02c	1.44 ± 0.03bc	1.43 ± 0.02bc	1.44 ± 0.02bc	1.45 ± 0.02b
Total P (g/kg)	1.37 ± 0.03a	1.34 ± 0.02ab	1.33 ± 0.03bc	1.29 ± 0.02c	1.25 ± 0.02d	1.26 ± 0.01d	1.25 ± 0.02d	1.24 ± 0.03d
Total K (g/kg)	3.95 ± 0.03a	3.31 ± 0.02e	3.37 ± 0.05d	3.38 ± 0.03d	3.39 ± 0.02d	3.50 ± 0.05c	3.81 ± 0.03b	3.82 ± 0.04b
Available N (mg/kg)	116.04 ± 0.17a	106.31 ± 0.14f	107.33 ± 0.24e	107.23 ± 0.30e	108.10 ± 0.35d	108.35 ± 0.39d	109.58 ± 0.48c	111.01 ± 0.37b
Available P (mg/kg)	42.13 ± 0.36a	31.21 ± 0.34f	34.27 ± 0.26e	35.44 ± 0.16d	36.99 ± 0.11c	37.14 ± 0.18c	37.32 ± 0.40c	38.48 ± 0.17b
Available K (mg/kg)	51.07 ± 0.42a	28.38 ± 0.29 g	29.03 ± 0.12f	30.39 ± 0.17e	31.75 ± 0.18d	32.26 ± 0.41c	33.02 ± 0.37b	33.19 ± 0.37b
Exchangeable Ca (mg/kg)	3015.05 ± 17.01a	2688.22 ± 1.96f	2714.75 ± 4.61e	2726.08 ± 4.27de	2736.86 ± 7.79d	2730.49 ± 9.91d	2770.54 ± 7.83c	2895.23 ± 16.44b
Exchangeable Mg (mg/kg)	206.62 ± 3.01a	186.92 ± 2.06d	187.80 ± 2.04d	191.37 ± 2.11c	195.15 ± 1.32b	195.55 ± 0.63b	198.09 ± 1.37b	198.19 ± 2.16b
Available Cu (mg/kg)	9.45 ± 0.30a	9.01 ± 0.31b	8.25 ± 0.22c	8.18 ± 0.13c	7.98 ± 0.17c	8.01 ± 0.15c	7.51 ± 0.12d	7.51 ± 0.10d
Available Zn (mg/kg)	7.92 ± 0.15a	7.69 ± 0.21a	7.26 ± 0.21b	6.93 ± 0.18c	6.83 ± 0.13c	6.82 ± 0.12c	6.48 ± 0.15d	6.46 ± 0.19d
Available Mn (mg/kg)	11.47 ± 0.33a	9.03 ± 0.13d	9.08 ± 0.12 cd	9.36 ± 0.14 cd	9.40 ± 0.25c	9.42 ± 0.22c	10.35 ± 0.25b	10.43 ± 0.26b
Available B (mg/kg)	0.71 ± 0.02a	0.43 ± 0.02e	0.47 ± 0.03d	0.54 ± 0.03c	0.55 ± 0.03c	0.55 ± 0.03c	0.57 ± 0.02c	0.66 ± 0.03b
Available Fe (mg/kg)	34.05 ± 0.55a	32.07 ± 0.56b	30.62 ± 0.43c	29.17 ± 0.33d	23.99 ± 0.44e	23.83 ± 0.37e	23.56 ± 0.29e	23.42 ± 0.26e

Data are expressed as mean ± standard deviation. Differences were tested using One-Way ANOVA followed by LSD test. Different letters indicate significant difference (P < 0.05) between treatments.

Both exchangeable Ca content and Mg content of the soil in mile-a-minute monoculture were significantly greater (P < 0.05) than those of sweet potato in monoculture, and significantly decreased (P < 0.05) as the proportion of sweet potato increased in mixed culture (Table 3). For the soil micronutrients in monoculture, available Cu, available Zn and available Fe associated with soil where mile-a-minute was grown were all significantly less (P < 0.05) than those of sweet potato, and increased as the proportion of sweet potato increased in mixed culture. However, available Mn content and B content of mile-a-minute were significantly greater (P < 0.05) than that of sweet potato in monoculture, and gradually decreased as the sweet potato proportion increased in mixed culture.

## Discussion

In the process of biological invasion, invasive alien plants may alter both the structure and function of ecosystems owing to their high degree of adaptability, morphological plasticity, competitive ability and potential to modify soil properties [10,13,32,33]. During interspecific competition, morphological characteristics and biomass tend to be the most important measured indices [10,13], and compared to native species, invasive plant species usually have greater morphological plasticity and biomass. However, our research found that morphological and biomass characteristics of mile-a-minute put it at a disadvantage when grown in association with sweet potato. In mixed and monoculture, the total biomass per sweet potato plant was significantly greater (P < 0.05) than that of mile-a-minute for ratios of sweet potato to mile-a-minute of greater than 1:3. Because the initial size and weight of mile-a-minute and sweet potato plants were similar and they were grown under similar conditions, differences in final biomass were due to competiveness and plant morphology. The relative yield (RY) and relative yield total (RYT) of mile-a-minute and sweet potato were significantly less (P < 0.05) than 1.0 in mixed culture, indicating that intraspecific competition was less than interspecific competition. The competitive balance index (CB index) of sweet potato and mile-a-minute was significantly greater than zero (P < 0.05) and positively correlated (P < 0.01) for a ratio of sweet potato to mile-a-minute of greater than 1:3, indicating that sweet potato had higher competitive ability than mile-a-minute. Similarly, sweet potato was found to significantly reduce population density and importance values of invasive alien species *Ageratum conyzoides*, *Bidens pilosa*, *Eleusine indica*, and *Galinsoga parviflora* and native species, *Digitaria sanguinalis* and *Portulaca oleracea* in China [34]. Sweet potato was also found to be highly competitive with various weed species in South Carolina [35].

Both sweet potato and mile-a-minute are perennial evergreen vines that share many morphological similarities [30], occupying virtually the same niche when grown in agricultural land in prostrate form. Mile-a-minute exhibits a high degree of morphological plasticity and has a large capacity for asexual propagation [26,27]. Sweet potato likewise exhibits a high capacity for asexual reproduction, indicated by the fact that most local villagers only use its root and stem for cultivation. The present study showed that flowering of mile-a-minute was significantly suppressed (P < 0.05) in mixed culture, and that the inhibition rates were higher than those of sweet potato with decreasing proportions of mile-a-minute. The ability to suppress seed production is important in terms of reducing the potential for rapid population growth of mile-a-minute [22].

In mixed culture, the main stem length, branch length, and internode length of mile-a-minute were significantly suppressed (P < 0.05) with increasing proportions of sweet potato. The internode length of mile-a-minute is greater than that of sweet potato, but the shorter internode length of sweet potato enables it to rapidly multiply internode number to facilitate soil contact and better access to soil nutrients. This relationship was further confirmed by observing the number of adventitious roots and biomass of mile-a-minute and sweet potato in mixed culture. Tillering or branching is an important means to compete with other plants, and also has been considered as a means of pre-empting resources during scramble competition [10]. In monoculture, the branch number of mile-a-minute was greater than that of sweet potato*;* however, in mixed culture its branch number was obviously suppressed, and the branch number of sweet potato was usually increased, to the detriment of mile-a-minute. By contrast, studies of competition with other types of weeds found that a sweet potato cultivar with a sprawling growth form did not compete as well as a more erect cultivar [35], illustrating the importance of understanding the specifics of particular competitive interactions.

Leaf area provides a major index to measure growth condition and solar energy utilization efficiency of plants [36]. Xu *et al*. [37] reported that mile-a-minute seedlings were inhibited by the aqueous leaf extract of sweet potato, with higher extract concentrations causing progressively stronger inhibition of mile-a-minute. The present research found that in all treatments, both the leafstalk length and leaf area of mile-a-minute were less than that for sweet potato*.* In monoculture, leafstalk length and leaf area of mile-a-minute were only 41% and 21% of that of sweet potato, respectively, and in mixed culture, both leafstalk length and leaf area of mile-a-minute were reduced with increased proportions of sweet potato. In mixed culture, 70-90% of mile-a-minute stems and leaves were covered by sweet potato, leading to a serious decline in mile-a-minute biomass.

Successful invasive plants may alter soil conditions such as nutrient availability, microbial composition and functioning, and in turn the altered soil conditions in some invaded ecosystems may promote further invasion [38]. Recent studies indicated that mile-a-minute modified the soil microbial community structure and soil chemical properties, possibly creating soil conditions that favor it over native plants [19,38,39]. Our findings found that with the exception of pH, soil nutrient contents of initial soil (CK) were significantly higher than contents following the seven treatments, demonstrating that both of sweet potato and mile-a-minute deplete soil nutrients during their growth. The concentrations of most soil macro-nutrients and secondary soil elements were significantly greater (P < 0.05) in mile-a-minute monoculture soil than in sweet potato monoculture soil, and were reduced by the competition of sweet potato in the mixture, indicating that sweet potato has a stronger capacity to consume nutrients than mile-a-minute. Furthermore, soil nutrients absorbed by mile-a-minute were greatly reduced when the plant was grown in mixed culture with sweet potato*.*

Compared with mechanical or chemical control, replacement control clearly has the potential to provide a more sustainable management option for growers, as seen in the present study and other related studies [9,10]. Utilizing alternative crops in this way serves to increase crop diversity, creating a more resilient system more similar ecologically to natural, more stable systems [2-4,6]. Furthermore, in the case of mile-a-minute, mechanical or chemical control often proves difficult or even counterproductive, resulting in limited options for managers [14,24]. This difficulty extends to natural areas, so there might well be a role for planting sweet potato or perhaps a native plant species with competitive abilities comparable to sweet potato in natural areas infested by mile-a-minute. As mentioned previously, competition between locally available plants and mile-a-minute has recently been investigated in China [9,29-31], and if the present study is any indication, there may be numerous opportunities to utilize plant competition in a variety of crop and non-crop situations. Although mile-a-minute is capable of rapid growth due to high photosynthetic rates at high light intensities, this capability is markedly reduced under shaded conditions, making it vulnerable to competition [40]. This “Achilles heel” that makes mile-a-minute vulnerable to replacement control may well be a weakness in many other disturbance-adapted weeds that may be overlooked by managers who assume that control must exclusively rely on mechanical or chemical control, or for that matter, biological control by pathogens or insects.

## Conclusion

The competitive advantage of sweet potato over mile-a-minute in terms of both plant growth and nutrient utilization that we observed could be used to reduce mile-a-minute growth in tropical and subtropical agricultural regions suitable for cultivation of sweet potato. At the same time, other techniques would be necessary to contain the spread of mile-a-minute in nearby natural areas, perhaps involving similar measures such as planting native vegetation that is competitive with mile-a-minute. During the growth of a mixed culture of mile-a-minute and sweet potato*,* sweet potato consumed more soil organic matter, total K, total N, available N, available P, available K, exchange Ca, exchange Mg, available Mn, and available B; meanwhile soil nutrients absorbed by mile-a-minute were considerably reduced. In order to provide a more comprehensive perspective on long-term management of mile-a-minute via competition with sweet potato, long-term successional patterns, growth-stage specific competition, and impacts of varying fertilizer levels and other environmental factors on the relationship between the two species should be researched further. This study also shows the potential value of replacement control methods which may apply to other crop-weed systems or invaded natural ecosystems.

## Methods

### Study site

The study site was located in Longchuan County (24°08′-24°39′ N, 97°17′-97°39′ E), Dehong Prefecture, in the northwest end of Yunnan Province. This area is characterized by a typical tropical climate, having a rainy season featuring heavy rainfall with 90% humidity alternating with a dry season. Rainfall averages 1595 mm per year and the annual mean temperature is 18.9°C [30]. In recent years the range of mile-a-minute has been expanding rapidly within Longchuan County, invading agricultural areas and forest margins.

### Study species

Mile-a-minute is one of the most serious invasive alien species in Dehong Prefecture where this study took place. This perennial weed exhibits a climbing growth form in forests, orchards and shrublands, but on roadsides, in wastelands, and other areas without woody vegetation, it takes on a prostrate form. It has infested sugarcane, orange, banana, coffee, pineapple, bamboo, sweet potato, maize crops, as well as artificial pasture and secondary forest in Longchuan County, Dehong Prefecture [17]. Mile-a-minute can invade disturbed environments via light weight seeds that are produced in great numbers, e.g. 170,000 m^2^ [22]. Spread is also facilitated by rooting of stem fragments; at a local level vegetative reproduction is responsible for most population growth [41].

Sweet potato, native to the American tropics, is one of the main food and cash crops in tropical and subtropical regions of Yunnan Province. It is also grown in many other regions of China and other subtropical or warm-temperate regions of the world as a food source. In Longchuan County, local villagers have grown it for over 100 years [30]. This herbaceous perennial vine usually exhibits a prostrate growth form in agricultural areas, so its niche is similar to that of mile-a-minute. Because of its purple root, it is also known as purple sweet potato. The aboveground parts of the plant are used for livestock fodder, and its roots are used for human consumption. It is propagated by seed or by clonal means, with 20–50 cm fragments with 3–5 nodes typically planted [42].

### Experiment design and data collection

The experiment was conducted during the April-December 2013 growing season within maize and sweet potato intercropping land in the vicinity of Zhangfeng Town, Longchuan County, Dehong Prefecture, utilizing a de Wit replacement series method [43]. On 15 April 2013, whole mile-a-minute plants (including roots) were collected from a mile-a-minute population located in a nearby forest margin and whole sweet potato plants were collected from farmland near Zhangfeng Town, respectively. To ensure relative uniformity among the experimental stock, one-node segments (fresh weight 3.0-3.5 g, 7–8 cm pieces) were taken from central stem portions of relatively young plants of similar size from both species. All materials were placed in Hoagland’s solution [44] and grown for 10 days. On 25 April 2013, the sprouts derived from cuttings of both species were transplanted in the field test plots. Seven ratios of sweet potato and mile-a-minute plants were utilized (4:0, 3:1, 2:1, 1:1, 1:2, 1:3, 0:4) while maintaining a constant planting of 20 plants m^−2^ (0.25 m × 0.20 m space). All plots were arranged in a complete randomized design with 4 replicates utilizing 25 m^2^ plots (5 m × 5 m). All plants were distributed evenly within the plot. During the experiment, the two species exhibited prostrate growth. The plots were not weeded and no synthetic fertilizers were used.

The experiment ended on 15 December 2013, 8 months after planting. Thirty five plants of each species were selected randomly and harvested within the middle region of each plot. Mile-a-minute and sweet potato plants were carefully removed, separated, and weighed. Total shoot length, main stem length (for mile-a-minute, after the one-node cutting with two leaves produced two main stems from each sprout; for sweet potato after the one-node cutting with one leaf produced one main stem from each sprout), branch length, internode length, branch number, leafstalk length (just the petiole, not including leaf blade), leaf area, and number of flowers per shoot were counted and measured. Here, we did not measure seed characteristics (size, length and biomass) because at this point in time both species were still growing vigorously; after flowering mile-a-minute tended to wither whereas sweet potato continued to grow. Branch length was measured as the sum of sub-branches coming off the main stem. Leaves were clipped and passed through a leaf-area meter (Li-3000A; Li-Cor Corp.) to determine leaf area index. Then roots were rinsed gently with water to remove soil particles. The adventitious root (produced by stems aboveground) weight and total biomass of each plant were measured after drying for 72 h at 78°C (0.001 g).

To examine the effects of the interaction of the two plant species on soil traits, initial soil samples from experimental units and soil samples (0–10 cm in depth) after harvest were collected from each of the 25 m^2^ plots. Fifty soil samples were taken randomly from each plot and then combined and treated as one composite sample. Soils were characterized by measuring the pH, soil organic matter, total and available N, total and available P, total and available K, exchange Ca and Mg, and available Cu, Zn, Fe, Mn, B at the Soil Analysis and Detection Center of Agricultural Environment and Resource Research Institute, Yunnan Academy of Agricultural Sciences, China.

### Data analyses

Relative yield per plant (RY) [43], relative yield total (RYT) [45] and Competitive Balance index (CB) [46] were calculated from final biomass (dry weight) for each species in each plot. These measures provide information on the competitive interaction between species in a mixed culture by comparison to growth in monoculture.

Relative yield per plant of species a or b in a mixed culture with species b or a was calculated as:

$$ \mathrm{R}{\mathrm{Y}}_{\mathrm{a}}={\mathrm{Y}}_{\mathrm{a}\mathrm{b}}/{\mathrm{Y}}_{\mathrm{a}}\mathrm{or}\ \mathrm{R}{\mathrm{Y}}_{\mathrm{b}}={\mathrm{Y}}_{\mathrm{b}\mathrm{a}}/{\mathrm{Y}}_{\mathrm{b}} $$

Relative yield total was calculated as:

$$ \mathrm{R}\mathrm{Y}\mathrm{T}=\left(\mathrm{R}{\mathrm{Y}}_{\mathrm{ab}}+\mathrm{R}{\mathrm{Y}}_{\mathrm{ba}}\right)/2 $$

Finally, competitive balance index was calculated as:

$$ \mathrm{C}{\mathrm{B}}_{\mathrm{a}}=\mathsf{In}\left(\mathrm{R}{\mathrm{Y}}_{\mathrm{a}}/\mathrm{R}{\mathrm{Y}}_{\mathrm{b}}\right) $$

Where Y_ab_ is the yield for species a growing with species b (g/individual), Y_ba_ is the yield for species b growing with species a, Y_a_ is the yield for species a growing in pure culture (g/individual), Y_b_ is the yield for species b growing in pure culture.

RY_ab_ measures the average performance of individuals in mixed cultures compared to that of individuals in pure cultures. A RY_ab_ of 1.00 indicates species a and b are both equal in terms of intraspecific competition and interspecific competition. A RY_ab_ greater than 1.00 means intraspecific competition of species a and b is higher than interspecific competition, and a RY_ab_ of less than 1.00 implies intraspecific competition of species a and b is less than interspecific competition. RYT is the weighted sum of Relative Yields for the mixed culture components. A RYT of 1.00 means that both species are competing for the same resources, and one is potentially capable of excluding the other; a RYT of greater than 1.00 means that the two species exploit different resources and therefore do not compete (e.g., due to different root depths); finally, an RYT of less than 1.00 implies that the two species are mutually antagonistic, with both having a detrimental effect on the other [45]. Values of CB_a_ greater than 0 indicate that species a is more competitive than species [46].

All growth variables (shoot length, branch number, leaf area, flower number, and dry weight biomass) of both plant species, and soil properties were analyzed by analysis of variance (one-way ANOVA). If significant differences were detected with the ANOVA, Duncan’s multiple range tests were used to detect differences among treatments at a 5% level of significance. RY and RYT from each mixed culture were compared to the value of 1.00 using t-tests (*P* = 0.05 or *P* = 0.01), and values of RYT were tested for deviation from 1.0 and values of CB for deviation from 0 using a paired *t*-test.

### Availability of supporting data

The data set supporting the results of this article is available in the Dryad Digital Repository http://dx.doi.org/10.5061/dryad.vb1qv [47].
